# Cases of Paralysis of the Vesica Urinaria, and Ruptures of the Same Organ from Distention

**Published:** 1801-07

**Authors:** Thomas Purton

**Affiliations:** Member of the Royal College of Surgeons in London


### Medical and Phyſical Journal.

VOL. VI.] JULY, 1801. [NO. XXIX.

### *Caſes of Paralyſis of the Veſica Urinaria, and Ruptures of the ſame Organ from Diſtention; by* THOMAS PURTON, Member of the Royal College of Surgeons in London.

To the EDITORS of the MEDICAL and PHYSICAL JOURNAL.

Gentlemen,

THE injuries which the bladder ſuffers from the neglect or ignorance of the uſe of the catheter, if I may judge from the caſes which have come under my own knowledge, are by no means infrequent. It is a circumſtance which cannot poſſibly take place under the attendance of a cautious and experienced practitioner; therefore, whenever the bladder ſuffers from an injury of this nature, it muſt be either from *neglect* or *ignorance.*

The caſe of Paralyſis was in a young woman, who had been delivered by a midwife of her firſt child, after a very lingering labour; this happened on a Friday, and I ſaw her on the following Sunday, when I was informed that ſhe had not paſſed any urine ſince the Wedneſday preceding her delivery, although I was told by the attendants, that *very powerful things had been given*, by the directions of the midwife, *to force her water.* I found her abdomen, of courſe, uncommonly diſtended, with much pain and conſiderable fever: I immediately introduced the catheter, and drew off the quantity of *nearly two gallons* of high coloured urine, which operation I was obliged to repeat every night and morning for three weeks, before the muſcular fibres of the bladder were reſtored to their former healthy action.

The ſubject of the next caſe was the mother of many children. In her laſt labour, which was very lingering, the head of the child preſſed ſo long upon a diſtended bladder, that it produced a ſphacelus, and the contents were evacuated per vaginam ſoon after her delivery. This woman is ſtill living; but but from the continual dribbling of the urine, and the consequent irritation, life is become a burthen to her.

The third case was that of a poor woman, who, after a very lingering and laborious labour, was delivered of her first child with great difficulty by the forceps. This woman also, from an unaccountable mistake in the practitioner who attended, was under a course of diuretics for two days after her delivery, thus pouring more into an already distended, and, probably, inflamed bladder, until a mortification and sloughing of its coats took place, which might possibly have been prevented by a more judicious treatment. The statement of this poor woman's case, which I received from a very respectable gentleman, who was afterwards called on to attend her, was very deplorable: In consequence of neglect, probably, the earthy part of the urine was suffered to deposit itself, so as almost to close the entrance of the vagina. On examination, the state of the parts was truly formidable; the finger was passed with difficulty through a rocky canal, surrounding the os externum, and extending some way up the vagina: this extraneous matter produced an extensive ulceration, which, as it increased very rapidly, caused an insupportable irritation; however, by the persevering attention and care of this able practitioner, she was restored as far as art could accomplish or nature admit; so that this gentleman has attended her since in several labours, and one of the times she was delivered of twins.*

Two very useful inferences I think may be drawn from the preceding cases; the first is, the absolute necessity of students sparing no time nor expence in acquiring a perfect knowledge of every branch of their profession, before they attempt to practice for themselves, as I have known myself, more than one who has had the effrontery to set up his standard in a country village as "*Surgeon and Man-midwife*," with a knowledge little superior to an apothecary's apprentice. Secondly, the necessity of the attention which practitioners should pay to their patients making water during their labour, and to use the catheter as soon as there is the least call for it, lest they subject them to pain and misery for the remaining part of their lives; this is a circumstance which requires particular attention, previous to the use of other instruments. Another thing worthy of notice in the preceding case of paralysis, is the vast quantity of water that was drawn off, which renders it truly astonishing,

* Medical and Physical Journal, Vol. II. p. 153.

aſtoniſhing, that a rupture of the bladder did not alſo in that inſtance take place.

Since the above was drawn up for publication, another caſe of paralyſis, ſimilar to that already ſtated, has come under the obſervation of an intimate friend of mine; where the gentleman who firſt attended was either ſo ignorant of the nature of the complaint, (which would appear remarkable, as the woman had not paſſed any urine for four days after her delivery) or incompetent to the uſe of that ſimple inſtrument the catheter. Here, then, is another caſe which ſhews the abſolute expediency of a regular acquirement of every branch of our profeſſion, and not as many gentlemen, who to my knowledge have had the boldneſs to ſettle, without either the benefit of an hoſpital education, or even attending one ſingle lecture on any branch of the medical department. This practice is becoming *so general*, that it ſurely calls for *legislative interference. No person ought to be allowed to settle, unleſs he could produce the proper certificates from the different seminaries.*

The ignorant man, under the preſent regulation, if he is poſſeſſed of a *certain species of plausibility*, may have an equal, if not a ſuperior advantage to the experienced practitioner; for unleſs there was ſome *test devised*, ſo that *ignorance* could be *fully exposed*, a man's abilities, however eminent, may by chance and ill fortune be overlooked, when one, without a grain of practical knowledge, may be preferred before him: for I think every gentleman will coincide with me in opinion, that it is virtually impoſſible that any perſon but a medical man *can judge* of a medical man's abilities.

I am, &c.

*Alcester, June* 6, 1801. T. PURTON.

